# A Novel Approach for Determining the Critical Quality Attributes of Mesenchymal Stem Cells by Specifying Cell Population With Replication Potential

**DOI:** 10.1093/stcltm/szad005

**Published:** 2023-03-14

**Authors:** Takako Yamamoto, Mao Arita, Takashi Tamura, Miho Saito, Hirohito Katayama, Hirotaka Kuroda, Takashi Suzuki, Shin Kawamata

**Affiliations:** R&D Center for Cell Therapy, Foundation for Biomedical Research and Innovation, Kobe, Japan; R&D Center for Cell Therapy, Foundation for Biomedical Research and Innovation, Kobe, Japan; R&D Center for Cell Therapy, Foundation for Biomedical Research and Innovation, Kobe, Japan; R&D Center for Cell Therapy, Foundation for Biomedical Research and Innovation, Kobe, Japan; Novartis Pharma KK, Tokyo, Japan; Shimadzu Corp., Kyoto, Japan; Graduate School of Engineering, Osaka University, Osaka, Japan; Shimadzu Corp., Kyoto, Japan; R&D Center for Cell Therapy, Foundation for Biomedical Research and Innovation, Kobe, Japan

**Keywords:** mesenchymal stem cells, mitochondrial function, critical quality attribute, critical process parameter, Redox system

## Abstract

We introduce a novel approach to determine the critical quality attributes (CQAs) of mesenchymal stem cells (MSCs) expected to exert immunosuppressive effects. MSCs retained homeostatic replication potentials, such as sustainable growth and consistent cell morphology as a population, in early passages, but lost them in late passages. Characteristic surface markers of MSCs (ie, CD73, CD90, and CD105) were no longer expressed at 2 weeks after subcutaneous transplantation into NOG mice when MSCs from late passages were transplanted, but not when MSCs from early passages were transplanted, suggesting that the biological effects of the MSCs differed according to the timing of cell harvesting and highlighting the importance of specifying MSCs that retained homeostatic features to define the CQAs. The homeostatic features of MSCs related to the balance of the redox system, nutrient requirements, and mitochondrial function were also observed until a certain passage. Therefore, we could define the CQAs of MSCs related to manufacturing by selecting process parameters (PPs) underlying the homeostatic features of MSCs and measuring these PPs quantitatively to specify the cell population with homeostatic features by limiting the passage number. The validity of the PPs stipulated in our pilot study was verified using an SKG murine arthritis model, and critical PPs (CPPs) were then selected among the PPs. Thus, CQAs related to manufacturing in the developmental phase could be defined by the CPPs in this manner, and the concept of CQAs could be refined continuously toward commercial manufacturing.

## Graphical Abstract

**Graphical Abstract:**
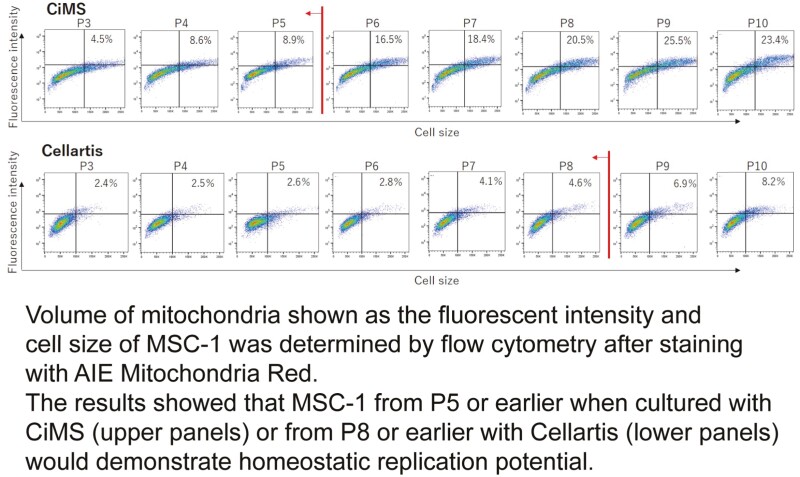

**Significance Statement**

We propose a novel approach to determining the critical quality attributes (CQAs) of mesenchymal stem cells (MSCs) related to manufacturing by specifying the cell population retaining homeostatic features, including sustainable growth rate, cell morphology consistency as a population, balance in the redox system, stable nutrient requirements and persistent mitochondrial function, harvesting cells with process parameters underlying these features and examining the pharmacological potency of the harvested cells with in vitro assays. The validity of the process parameters was confirmed using a murine arthritis model.

## Introduction

Despite many mesenchymal stem cell (MSC)-based clinical studies and therapies targeting a variety of diseases, such as graft-versus-host disease after bone marrow transplantation,^1^ acute respiratory distress syndrome,^2^ coronavirus disease-2019,^3^ autoimmune diseases,^4^ arthritis,^5^ and inflammatory bowel disease,^6^ no consensus on the definition of MSCs has been reached.

In the last 2 decades, several attempts have been made to establish the critical quality attributes (CQAs) of MSCs. In 2006, the International Society for Cell Therapy^7^ proposed 3 minimal requirements for defining MSCs for research purposes: (1) MSCs must be able to adhere to plastic when maintained under standard culture conditions; (2) MSCs must express CD105, CD73, and CD90 but be negative for CD45, CD34, CD14 or CD11b, CD79α or CD19, and HLA-DR surface molecules; and (3) MSCs must be able to differentiate into osteoblasts, adipocytes, or chondroblasts in vitro. These definitions of MSCs may not be sufficient to develop new MSC-based cell therapies for regulatory applications and may not be suitable for manufacturing various MSC-based products with different biological activities.

In this study, we aimed to determine the CQAs of MSCs related to manufacturing by defining the indication of MSCs for anti-inflammatory effects.

## Results

### Identification of Cell Populations with Homeostatic Replication Potential According to Growth Rate

**Figure 1.**
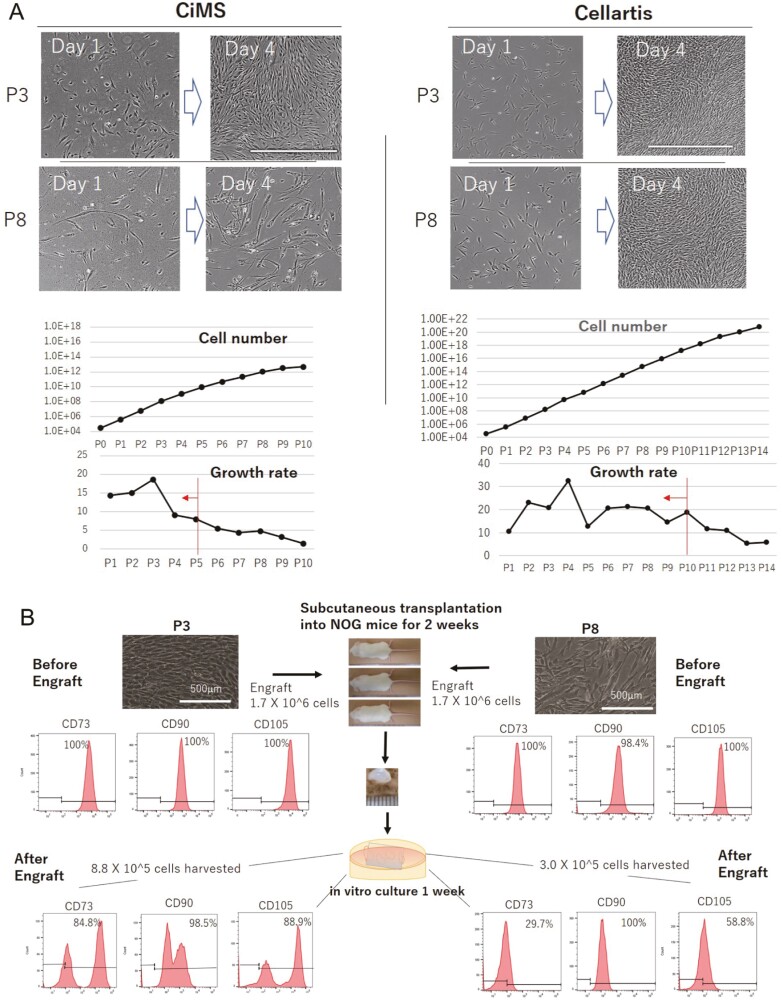

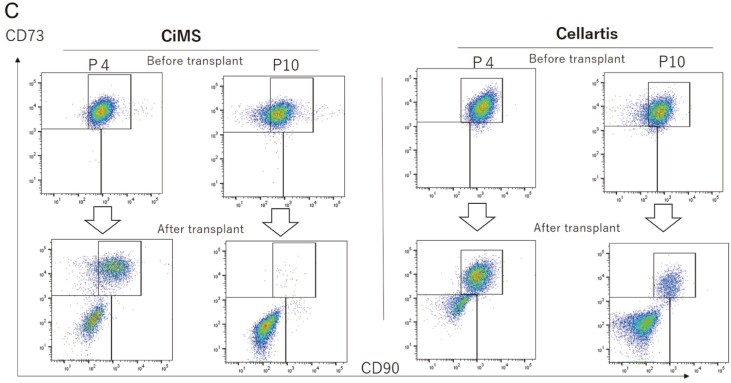
Determination of the target cell population according to consistent cell proliferation in culture. **A**: Photographs of MSCs cultured with CiMS for 1 or 4 days at P3 or P8 (left panels) or with Cellartis for 1 or 4 days at P3 or P8 (right panels). MSCs were seeded at 3 × 10^4^ cells/well in 6-well plates and then cultured with CiMS (left panels) or Cellartis (right panels) for 4 days before passaging. Scale bar: 1000 μm. The calculated cell number or growth rate was plotted against the passage (P) number in line graphs. The line and arrow indicate the critical passage number at which proliferation of the target cell population changed. **B**: Schema of transplantation of MSCs cultured at different passages into NOG mice. MSCs (1.7 × 10^6^) cultured with CiMS at P3 or P8 were transplanted subcutaneously into 3 NOG mice using Matrigel for 2 weeks. The transplanted cells were then removed from the mice and recovered in culture for 1 week. The expression of the MSC surface antigens CD73, CD90, and CD105 before and after transplantation was examined. Percentages of positively stained cells were added to the relevant histogram after assessment by flow cytometry. A representative result from three transplants is shown in the schema. **C**: Expression of CD73 and CD90 on the surface of MSCs cultured with CiMS at P4 or P10 before (upper left panels) and after transplantation (lower left panels) or with Cellartis at P4 and P10 (upper right panels) and after transplantation (lower right panels). Representative results from 3 transplants are shown.

MSCs in culture have a heterogenous morphology and growth rate depending on the MSC clone and culture protocol; however, the growth rate and composition of heterogenous cells in culture are fairly consistent during the early passages, regardless of the cell origin, clone, or culture medium (Fig. 1A; Supplementary Fig. S1, S2). This observation suggests that MSCs are stem cells of mesodermal origin with homeostatic replication potential, and such potential can be observed in all MSC clones during the early passages. When culturing MSC-1 (Lonza) with CiMS (Nipro) or Cellartis (Takara), sustainable growth of MSC-1 (more than 10-fold per passage) was observed until passage (P) 5 for MSC-1 cultured with CiMS or P10 for those cultured with Cellartis (Fig. 1A). This result suggested that the characteristic features of MSC-1, including efficacy, would change before and after P5 when cultured with CiMS or P10 when cultured with Cellartis. Moreover, the results highlighted the importance of searching for the PPs that could specify the target cell population retaining homeostatic replication potential multidirectionally through a series of pilot studies. This idea was supported by a mouse subcutaneous transplantation experiment using MSC. MSC-1 cultured with CiMS from P3 and a later passage (P8), at which time they showed a distinct morphology and growth rate, were subcutaneously transplanted into NOG mice (Fig. 1B). Although the expression profiles of CD73, CD90, and CD105 determined by flow cytometry before transplantation were almost identical, P8 cells had lost expression of these markers at 2 weeks after transplantation by 1 week of in vitro culture. This indicated that human MSCs distinguished by anti-human CD45 antibodies had differentiated into mesodermal cells other than MSCs, whereas cells from P3 partially maintained their MSC features. Thus, the efficacy of P3 and P8 MSCs may differ, and it may be necessary to define cell populations to address the biological activities of MSCs. Based on this pilot study, we carried out an additional pilot study. MSC-1 was cultured with either CiMS or Cellartis and transplanted subcutaneously into NOG mice at P4 or P10. Two weeks later, transplanted cells were recovered and subjected to ex vivo culture for 1 week. The expression of CD73 and CD90 was then examined (Fig. 1C). This experiment confirmed the results of the pilot study (Fig. 1B), showing that MSC-1 cultured with CiMS and Cellartis and harvested at P4 (supposed to be the target cells) retained the surface markers of MSCs, but lost them completely when cultured with CiMS and harvested at P10 (supposed to be off-target cells) and only partially retained them when cultured with Cellartis and harvested at P10 (target/off-target marginal cells). These pilot studies emphasized the importance of harvesting the target population to demonstrate the biological efficacy of MSCs and suggested that a growth rate of over 10-fold within one passage could be used as a PP. Furthermore, these findings showed that the threshold passage number may represent the boundary for harvesting target cells to retain biological activities after transplantation.

### Identification of Cell Populations with Homeostatic Replication Potential by Senescence-Related PPs

Continuous reduction of the cell growth rate could indicate that cells in culture are in a senescent state and not the target cell population. Although monitoring the cell growth rate is a simple and robust PP to address whether the cells are in a senescent state or not in cases of MSC manufacturing, the usability of other PPs related to senescence was examined.

**Figure 2.**
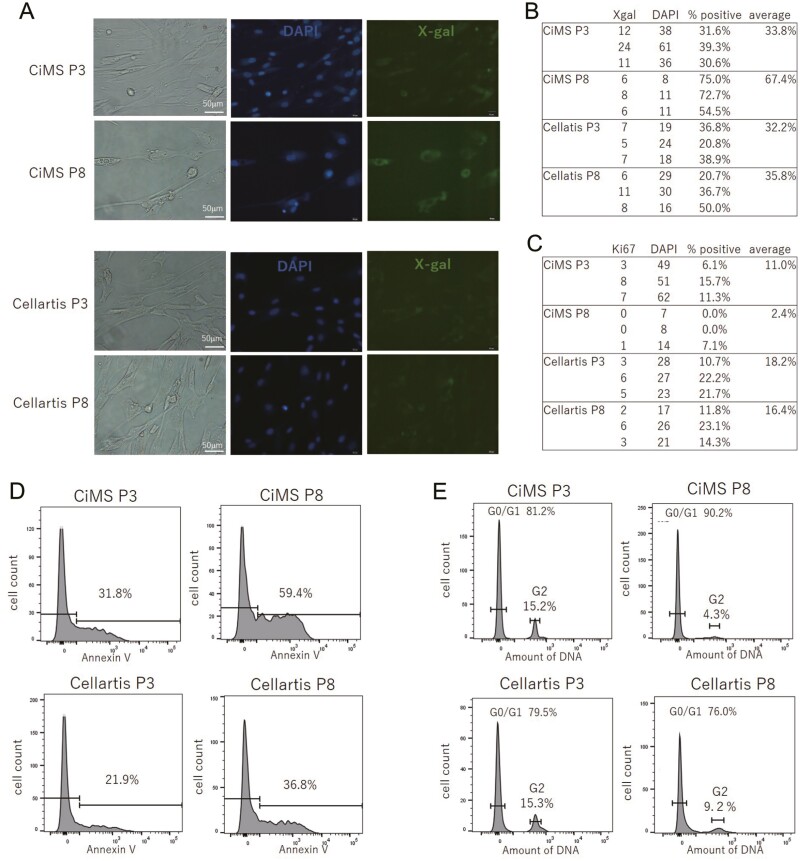
Cells in late passages ceased to proliferate and underwent apoptotic changes. MSCs were cultured with CiMS at P3 or P8 or with Cellartis at P3 or P8. **A**: Cells in the senescent state were positively stained with X-gal. **B**: The percentages of cells stained with X-Gal under different culture conditions were determined using Image J software and are shown in the table. **C**: Proliferating cells were positively stained with Ki67. The percentages of cells stained with Ki67 under different culture conditions are shown in the table. **D**: Cells that underwent apoptosis were positively stained with Annexin V. The percentages of cells stained with Annexin V under different culture conditions were determined by flow cytometry. **E**: The percentages of cells in G_0_/G_1_ or G_2_ phases under different culture conditions were determined by flow cytometry after staining with cell cycle assay solution. Representative results from 3 independent experiments are shown in A–D.

In this context, MSC-1 cultured with CiMS or Cellartis at P3 or P8 were stained with Ki67 (cell proliferation), X-Gal (aging), Annex V (apoptosis), or cell cycle assay solution blue for cell cycle analysis (cell proliferation).^8^ Notably, MSC-1 cultured with CiMS at a late passage (P8) showed marked X-Gal staining (67.4%) compared with cells at an early passage (P3; 33.8%) or cells cultured with Cellartis at P3 (32.2%) or at P8 (35.8%; Fig. 2A, 2B). These cells also showed a reduced percentage of Ki67-positive staining at P8 (2.4%), but not at an early passage (P3; 11.0%) or when cultured with Cellartis at P3 (18.2%) or at P8 (16.4%; Fig. 2C). Similar results were obtained for Annexin V staining, demonstrating apoptotic changes in cells and changes in cell cycle distribution to address cell proliferation. Namely, the percentage of cells positive for Annexin V staining among MSC-1 cultured with CiMS at a late passage (P8) was markedly high (59.4%) compared with that at an early passage (P3; 31.8%) or cells cultured with Cellartis at P3 (21.9%) or at P8 (36.8%). Furthermore, cell cycle analysis demonstrated that the G_0_/G_1_:G_2_ distribution ratio of MSC-1 cultured with CiMS at P8 was 90.2%:4.3% and that at P3 81.2%:15.2%, whereas those for cells cultured with Cellartis were 79.5%:15.3% at P3 and 76.0%:9.2% at P8. These findings suggested that the proliferation rate of MSC-1 cultured with CiMS at P8 was reduced compared with that at P3. This series of experimental results to address the senescent state of MSCs was consistent with the results of monitoring the cell growth rate, supporting the idea that verification of the nonattenuated cell growth rate could be a simple and robust CPP for harvesting MSC with homeostatic replication potential.

These findings also suggested the need to explore multidirectional PPs that can be exploited to harvest cells with homeostatic replication potential.

### Identification of Cell Populations with Homeostatic Replication Potential According to Cell Morphology Evaluation

**Figure 3.**
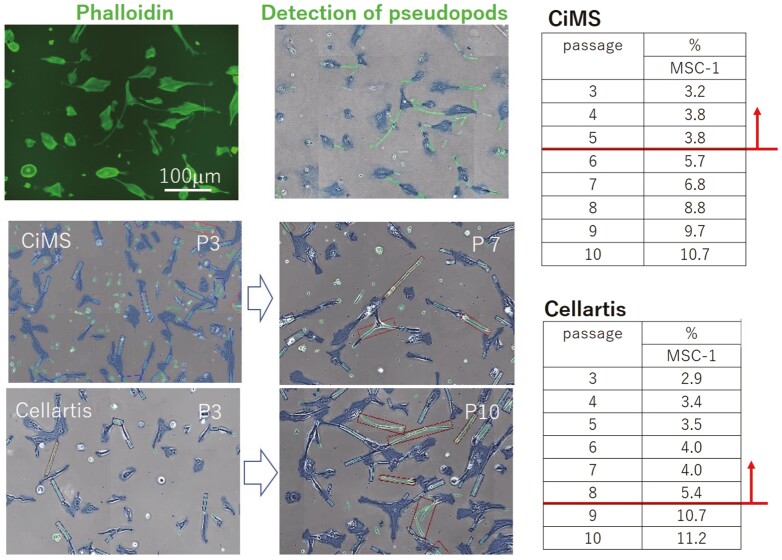
Determination of the target cell population according to the total area of the pseudopods of cultured cells. MSCs were stained with phalloidin to visualize the shape of cells, and the pseudopods were recognized as the green area with the morphology-recognizing function of Cell Pocket (left upper panels). The pseudopods of MSCs cultured with CiMS at P3 or P7 (left middle panels) or Cellartis at P3 or P10 (left bottom panels) were identified, and the total area of pseudopods of cells measured at P3-10 was calculated and shown as the percentage (%) of the pseudopod area relative to the total area of the cells in culture captured using Cell Pocket (right tables). The line and arrow indicate the critical passage number at which the pseudopod area of the target cell population changed. Representative results from 3 independent experiments are shown.

Based on the above results, another PP for harvesting the target cell population was a consistent cell morphology during passaging. Thus, we evaluated the morphology of individual cells by identifying pseudopods and measuring their lengths from several captured images using an artificial intelligence (AI)-based morphology recognizing system (Cell Pocket; Shimazu). Representative images used to identify and measure pseudopod length are shown in Fig. 3, and the threshold passage number at which the percentage of detected areas for pseudopods against those for total cell areas increased was evaluated. Using this method, homeostatic replication potential was assessed in terms of cell morphology changes. The percentage of the pseudopod area of MSC-1 was in the range of 3.2%-3.8% during P3-P5 but increased markedly after P6 (range: 5.7%-10.7% from P6 to P10) when cultured with CiMS. By contrast, when MSC-1 was cultured in Cellartis, the percentage of the pseudopod area increased, but remained in the range of 2.9%-5.4% during P3-P8 and increased markedly after P9 (range: 10.7%-11.2% from P9 to P10). Thus, homeostatic replication potential, measured by the length of pseudopods of cells in culture, decreased markedly at a certain passage, and the maximum permissible passage number to harvest the target cells was P5 for MSC-1 when cultured with CiMS and P8 for cells cultured with Cellartis (Fig. 3). The morphology of cultured cells varies with culture medium, cell origin, and cell clone, however, it is important to determine the threshold passage number at which homeostatic replication potential changes based on morphological assessment. Cell growth and morphology can be observed easily without the need for further analysis and destructive manipulation. However, these parameters are simply outcomes of the intracellular events occurring during a prolonged culture period and may not represent changes in the fundamental metabolic events related to maintenance of homeostasis or the homeostatic replication state. Therefore, the maximum passage number at which a target cell population should be cultured can be determined by considering other PPs related to the intracellular metabolic events.

Cell morphology is based on the dynamics of the cytoskeleton, including polymerization and depolymerization of stress fibers regulated by the balance between RhoA- and Rac-1-mediated signaling.^9,10^ Reactive oxygen species (ROS) produced by mitochondrial activation disrupts the balance between stress fiber polymerization and depolymerization,^11^ yielding spindle-shaped cells due to inhibition of stress fiber disassembly. This provides a rationale for measuring the balance of the redox system and mitochondrial activity as the PPs that underlie the formation of pseudopods in late passages.

### Identification of Cell Populations with Homeostatic Replication Potential According to Nutrient Requirements and the Metabolic Balance

**Figure 4.**
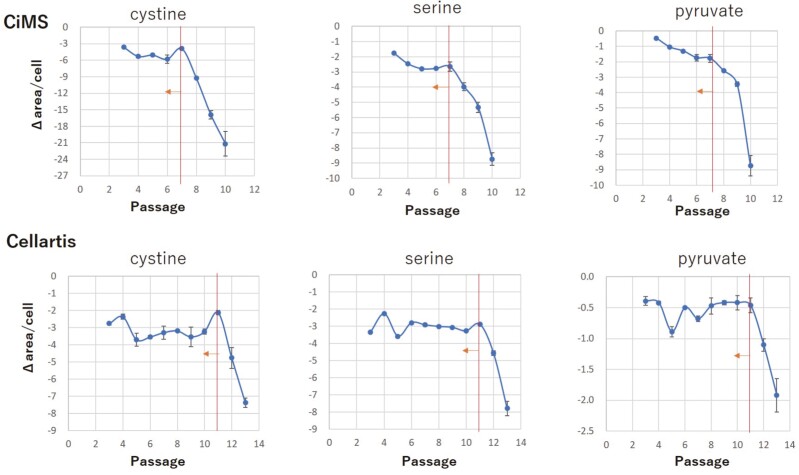
Determination of the target cell population according to mitochondrial activation measured by metabolic analysis. Metabolic analysis of MSCs cultured with CiMS (upper panels) or Cellartis (lower panels) at the designated passage (P) number was performed using LC-MS/MS. The absorption of cystine, serine, or pyruvate in the medium of MSCs compared with fresh medium without cells was measured and divided by the number of cells harvested at that passage and shown as the negative area ratio per cell (−Δarea/ cell). The line and arrow indicate the critical passage number at which the concentrations of cystine, serine, or pyruvate in the target cell population changed. Representative results from three independent experiments are shown.

MSCs are somatic cells that utilize ATP from mitochondria to maintain their biological activity; however, the activation of mitochondria is coupled with the generation of ROS, which must be disposed of via the redox system to maintain the metabolic balance within cells. The vigorous cell growth and high mitosis rate of early passage MSCs can lead to active replication of mitochondria and mitochondrial DNA and accumulation of ROS, which cannot be disposed of via the innate redox system, after a certain number of mitotic cycles.^12^ These events induce irreversible changes in the metabolic balance and the homeostatic reproduction potential of MSCs. Liquid chromatography-tandem mass spectrometry (LC-MS/MS) analysis of culture medium from MSCs grown in CiMS or Cellartis showed that the requirement for pyruvate in the tricarboxylic acid (TCA) cycle, and pyruvate and cystine used for the redox system from culture medium were dramatically increased at P7 and P11 when MSC-1 were cultured with CiMS and Cellartis, respectively (Fig. 4). These results supported that mitochondrial function may deteriorate progressively during the culture period but that a compensatory system, such as the redox system, may react to overcome mitochondrial dysfunction; however, such a system cannot be sustained and will fail after a certain passage. This failure will result in the sudden uptake of large amounts of pyruvate from the culture medium, causing inefficient ATP production and poor maintenance of the minimum biological activity of MSCs. The excessive uptake of cystine and serine from the culture medium observed after a certain passage number may indicate a direct disruption of the redox balance. Cystine is necessary for the synthesis of glutathione, a potent antioxidant, owing to its ability to remove ROS,^13^ and serine is a substrate of glycine, which is another component of glutathione.^14,15^ Excessive uptake of pyruvate, cystine, and serine into MSCs occurred simultaneously, suggesting that disruption of the redox system can lead to loss of metabolic balance and homeostatic replication potential. Thus, excessive uptake of pyruvate, cystine, and serine from the culture medium may distinguish cells with a metabolic imbalance from other cells. In the case of MSC-1, cells cultured with CiMS from P7 or earlier and those cultured with Cellartis from P10 or earlier showed consistent uptake of cystine, serine, and pyruvate and maintained their homeostatic replication potential. However, at later passages, these cells showed massive uptake of the corresponding amino acids, suggesting the loss of replication in these cells (Fig. 4).

Moreover, because the target amino acids to trace will differ according to the formula of the culture medium and to some extent the innate metabolic pathways of the specific MSC clone, the metabolic parameters that ensure homeostatic replication potential should be determined in a case-by-case manner depending on the protocol used.

### Evaluation of the Homeostatic Replication Potential of Cells According to the Mitochondrial Volume

**Figure 5.**
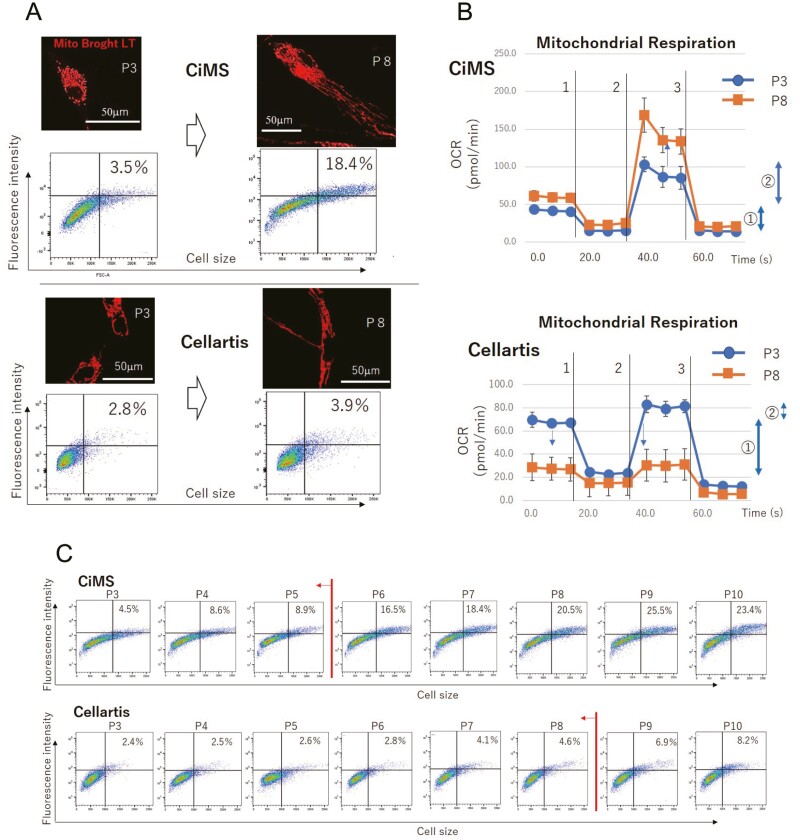
Determination of the target cell population according to the volume of the mitochondria. **A**: Cell size and mitochondrial morphology visualized by staining with AIE Mitochondria red. The volume of mitochondria from MSCs cultured with CiMS (upper panels) or Cellartis (lower panels) at P3 or P8 was determined by flow cytometry (shown as the fluorescence intensity). Representative results from 3 independent experiments are shown. **B**: Mitochondrial respiration of MSCs determined using a mitochondrial stress test kit. The oxygen consumption rate (OCR) of 4 × 10^4^ MSCs cultured with CiMS (upper panel) or Cellartis (lower panels) harvested at P3 or P8 after addition of (1) oligomycin, (2) carbonyl cyanide 4-(trifluoromethoxy) phenylhydrazone, (3) rotenin, and antimycin A is shown in the line graphs. In the graphs, ① indicates ATP production, ② indicates the spare respiratory capacity, and ①+② indicates the maximum respiration. Representative results from 3 independent experiments are shown. **C**: Volume of mitochondria in MSCs cultured with CiMS (upper panels) or Cellartis (lower panels) at P3-P10 was determined by flow cytometry (shown as the fluorescence intensity), and cell size was determined by staining with AIE Mitochondria red. The line and arrow indicate the putative passage number at which mitochondrial volume in the target cell population changes. Representative results from 3 independent experiments are shown.

Massive uptake of pyruvate during late passages suggests inefficient production of ATP, likely due to dysfunctional mitochondrial activity. Irreversible mitochondrial dysfunction occurs as a result of ROS accumulation during prolonged culture. Therefore, assessing mitochondrial function could provide a good PP to define cells with homeostatic replication potential. Notably, cell size is increased in later passages owing to de novo mitochondrial biogenesis and mitochondrial fusion, which is required to compensate for the function of deteriorated mitochondria and to produce the ATP required for the minimum biological activity of cells.^16,17^ The fusion of mitochondria in cells cultured with CiMS at P8, not with CiMS at P3 or Cellartis at P3 or at P8, was observed by staining mitochondria with Mito Brought LT (Fig. 5A). In this staining experiment, we observed a greater number of electrons anchored along the mitochondrial membrane as a result of mitochondrial biogenesis and fusion. Furthermore, the increase in cell size, which was necessary to accommodate the large volume of mitochondria, and the increased number of electrons along the mitochondrial membrane were observed simultaneously by flow cytometry after staining cells with AIE Mitochondria Red (Fig. 5A). The functions of mitochondria (eg, ATP production and spare respiratory capacity) during early and late passages were evaluated using a Seahorse XF Cell Mito Stress Test kit (Agilent) and measured as the oxygen consumption ratio (OCR, pmol/min). Cells cultured with CiMS did not produce ATP effectively, leaving a high spare respiratory capacity (P3 and P8), and the mitochondria had to increase in volume, both through fusion and biogenesis, to maintain the minimum ATP production requirements. This presumption was supported by the increased spare respiratory capacity at P8. Although cells cultured with Cellartis produced ATP quite effectively, leaving a lower spare respiratory capacity, the potential for ATP production was reduced during culture. However, mitochondrial fusion did not appear to have occurred, as no increase in the spare respiratory capacity was observed (Fig. 5B). Based on these findings, serial flow cytometric analyses were conducted to determine cell populations with homeostatic replication potential in terms of the degree of mitochondrial volume and cell size. The results showed that MSC-1 maintained both mitochondrial shape and cell size until P5 when cultured with CiMS and until P8 when cultured with Cellartis (Fig. 5C). These results suggested that measurement of mitochondrial volume and cell size simultaneously by flow cytometry could be applied as a PP to distinguish target cells from off-target cells. As shown in these experiments, mitochondrial function differed greatly according to the culture medium. Thus, the threshold passage number that could be used to distinguish the target cell population should be determined on a case-by-case basis using the procedures outlined in this report.

### Verification of the Stipulated PPs for Harvesting Target Cells Using Biological Efficacy Tests

Cell products are expected to exert immunosuppressive effects. Therefore, we next examined the pharmaceutical potency of the harvested cells based on the proposed PPs using in vitro assays. The potency of cells can be measured quantitively, eg, by analysis of the amount of released cytokines related to immune suppression and the percent induction of CD3^+^CD4^+^FOXP3 regulatory T cells (Tregs). Specifically, the immunosuppressive effects of MSCs were evaluated by assessing the release of interleukin (IL)-6^18^ and IL-10^19^ in the medium at 24 h (for analysis of acute reactions) or 72 h (for analysis of the effects on Treg induction) after coculturing MSCs with peripheral blood mononuclear cells (PBMCs) at a ratio of 1:20 in the presence of the T-cell-stimulating reagents phorbol 12-myristate 13-acetate (PMA) and ionomycin. The MSC: PBMC coculture ratio was determined based on the published therapeutic dose of MSCs administered to a patient weighing 60 kg.^20-22^ Because the results of in vitro assays may vary according to the PBMC lot used, the assay was repeated several times to confirm the robustness of cytokine release across different PBMC lots.

**Figure 6.**
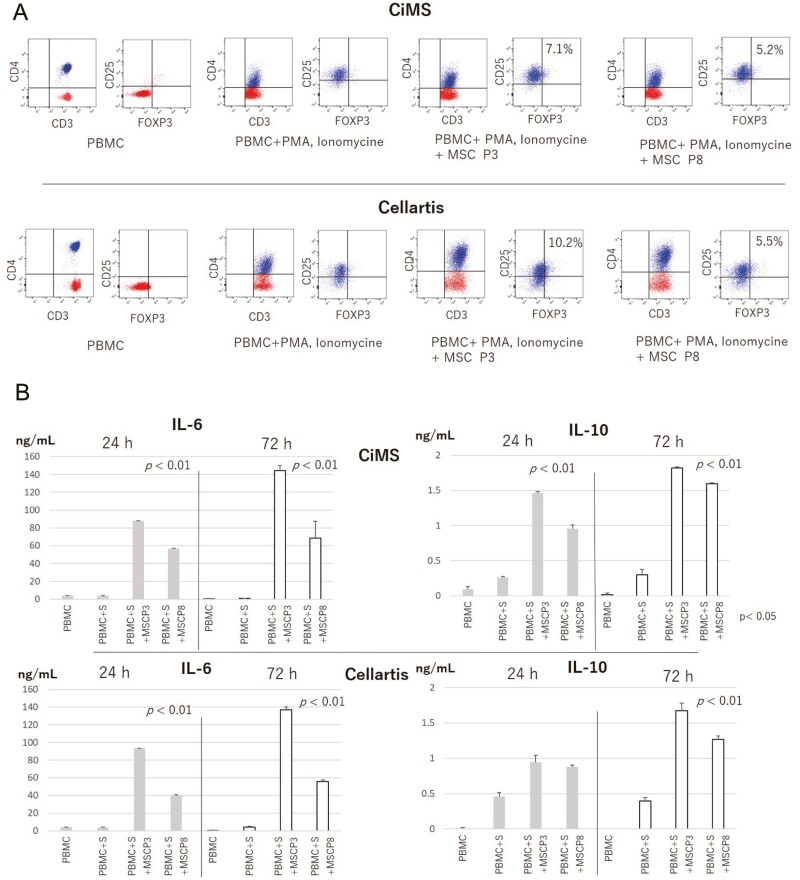

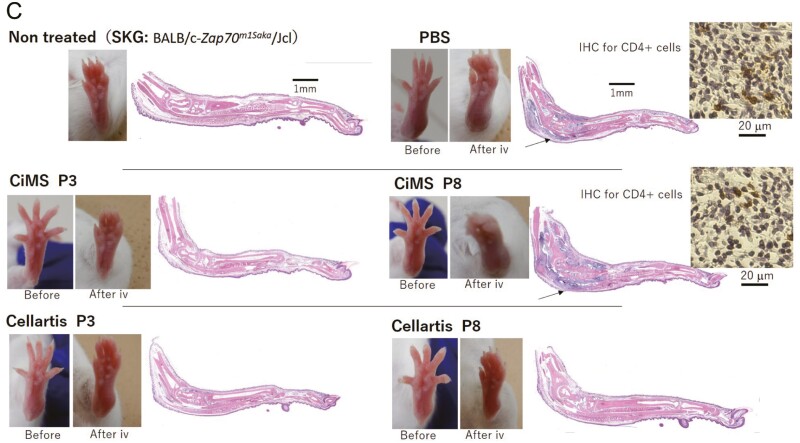
Demonstration of the in vitro and in vivo efficacy of MSCs from the target cell population. **A**: MSCs cultured with CiMS or Cellartis were harvested at P3 or P8 and cocultured with PBMCs at a ratio of 1:20. Induction of CD3^+^CD4^+^CD25^+^FOXP3^+^ Tregs in the absence or presence of PMA and ionomycin stimulation was evaluated by flow cytometry. Representative results from 3 independent experiments are shown. **B**: IL-6 and IL-10 secretion from MSCs. MSCs harvested at P3 or P8 after culture with CiMS (upper panels) or Cellartis (lower panels) were cocultured with PBMCs at a ratio of 1:20 in the absence or presence of PMA and ionomycin stimulation. After 24 or 72 h, cytokine secretion was measured by ELISA. *P* values for IL-6 or IL-11 secretion between at P3 and at P8 under different culture conditions are shown in the bar graphs. Representative results from three independent experiments are shown. **C**: MSCs harvested at P3 or P8 after culturing with CiMS or Cellartis were infused via the tail vein into SKG mice with mannan-induced arthritis. Tissue sections from the left hind legs of SKG mice administered PBS (top) or MSCs at P3 or P8 cultured with CiMS (middle) or Cellartis (bottom) are shown. Immunohistochemical staining using an anti-human CD4 antibody was performed using hind leg tissue sections from SKG mice with arthritis. Representative results from each treatment group (3 control mice, 5 MSC treated mice) are shown.

In our prior experiments using several PPs stipulated in a pilot study, we concluded that the maximum permissible passage number to harvest target cells with homeostatic replication potential for MSC-1 was P5 when cultured with CiMS and P8 when cultured with Cellartis from a risk management perspective. The pharmacological potency of cells harvested from different passage numbers was examined through a series of in vitro assays. Namely, MSC-1 cultured with CiMS at P3 (target cells) or P8 (off-target cells) or with Cellartis at P3 (target cells) or P8 (marginal, but target cells) were examined. Treg induction and IL-6 and IL-10 release at both 24 and 72 h after coculture of T cells with MSC-1 in the presence of PMA and ionomycin were observed, regardless of the use of the target cell or off-target cell population (Fig. 6A, 6B). However, Treg induction was more robust when T cells were cocultured with MSC-1 from P3 than when cultured with those from P8, suggesting that early passage MSCs (P3) may show more potent pharmacological effects and thus more potent immunosuppressive effects than cells from a later passage (P8) (Fig. 6A). Similarly, IL-6 and IL-10 release in coculture medium with MSC-1 from P3 was higher than that from P8 both at 24 and 72 h (*P* < .01, analysis of variance [ANOVA]), except for IL-10 release from MSC-1 cultured with Cellartis at 24 h (Fig. 6B). These findings suggested that the pharmacological effects of MSCs may be stronger at early passages than at later passages, regardless of the target cell or off-target cell population, highlighting the so-called quality versus quantity issue. Therefore, it is important to limit the permissible maximum passage number to harvest cells in a rational manner. Notably, in vitro potency assays may be necessary to address the pharmacological potency of the harvested cells and to reject the cells if no potency is observed, but may not be used to distinguish target cells from off-target cells. Assessment of the biological effects of target cells harvested based on the PPs and validation of the PPs selected in the pilot study can be performed using a mouse model of inflammatory disease.

SKG mice are BALB/c background mice with a point mutation in ZAP70, which is related to T-cell function. These mice exhibit arthritis-like inflammatory disease after intraperitoneal administration of mannan or β-glucan.^23^ MSCs cultured with CiMS at P3 (target cells) or P8 (off-target cells) or with Cellartis at P3 (target cells) or P8 (marginal, but target cells) were injected into SKG mice with arthritis via the tail vein at 6 weeks after mannan challenge. Tissue sections of the limb joints were obtained and evaluated 2 weeks later. The results clearly showed a therapeutic difference in immunosuppressive effects between the target (P3) and off-target cell population (P8) for cells cultured with CiMS (Fig. 6C; Supplementary Fig. S3 ). Furthermore, cells cultured with Cellartis and harvested at P3 (target cells) and P8 (marginal, but target cells) exhibited immunosuppressive effects after administration into SKG mice (Figs. 6C; Supplementary Fig. S3). These in vivo results verified the validity of the PPs selected in the pilot study and supported our approach of defining CQAs related to manufacturing in the developmental stage by specifying the target cells based on several selected PPs. The CPPs should be selected from the PPs extracted during pilot studies.

## Discussion and Conclusions

In this study, we developed an approach and procedure for defining CQAs in the developmental stage that could be applied to any MSC clone used for immunosuppressive therapy. In the first step, we characterized the cell population with homeostatic replication potential based on several biological features, including stable cell growth rate without tendency for attenuation, presence of cell morphology consistency as a cell population, balance of the redox system, stability of nutrient requirements, and persistent mitochondrial function without accompanying marked volume changes. Each parameter had a quantitative threshold that could be used to determine the maximum permissible passage number. Then, in vitro potency assays were developed to assess the pharmacological activities of the harvested cells. Finally, the biological effects of the harvested target cells and the validity of the stipulated PPs were verified using a mouse model of inflammatory disease.

For in vitro assays evaluating the pharmacological potency of the harvested cells, we measured several cytokines, including IL-2, IL-6, IL-10, IL-17, and transforming growth factor (TGF) β at 24 h (to analyze acute response) and 72 h (to analyze the effects on Treg induction) to select parameters for the efficacy assay. Induction of Tregs from PBMCs cocultured with MSCs for 72 h with PMA and ionomycin stimulation was also conducted in the in vitro assay. However, we did not observe any marked changes in the release of some cytokines, and we failed to obtain reproducible results among several pilot assays, with the exception of IL-6 and IL-10. IL-10 exerts anti-inflammatory effects,^24,25^ whereas IL-6 exerts various biological effects, both pro- and anti-inflammatory, via 3 different signaling pathways: classical (mediated by the membrane IL-6 receptor and gp130), trans (mediated by IL-6/soluble IL-6 receptor and membrane-bound gp130), and cluster (mediated by IL-6/membrane IL-6 receptor stimulating gp130 in neighboring cells) signaling depending on the administration method and clinical setting.^26^ The pro-inflammatory effects of IL-6 were demonstrated based on the therapeutic efficacy of the anti-IL-6 antibody tocilizumab^27^ for rheumatoid arthritis (RA); by contrast, IL-6 was reported to have anti-inflammatory effects in IL-6-knockout mice^18^ and to exert anti-inflammatory effects via trans signaling in severe chronic cutaneous graft-versus-host disease after allogenic cell transplantation.^28^ In particular, a study reporting that adipocyte-derived MSCs modulate myeloid cells to adopt an anti-inflammatory reparative phenotype via IL-6 and prostaglandin E2 secretion in MSC/PBMC cocultures^29^ supported our use of IL-6 release to evaluate the anti-inflammatory effects of MSCs. Further work is needed to improve the in vitro potency assay based on knowledge of the anti-inflammatory effects and mechanisms of MSCs.

## Materials and Methods

All experiments using human samples and animals were reviewed by the Institutional Review Board and the Animal Experiment Committee of the Foundation for Biomedical Research and Innovation.

### Cells and Cell Culture

The following MSC clones were obtained: MSC-1 (from adipocytes of a 42-year-old Hispanic female; Lonza, lot no. 0000669429), MSC-3 (from bone marrow of a 60-year-old Caucasian male; PromoCell, lot no. 4452012.1), MSC-4 (from adipocytes of a 58-year-old Caucasian male; Lonza, lot no. 0000672320), and MSC-7 (from umbilical cord matrix of a Caucasian female; PromoCell, lot no. 450Z018). The MSCs were cultured with CiMS (Nipro, Tokyo, cat. no. 87-070,072), Cellartis MSC Xeno-Free Culture Medium (Takara; cat. no. Y50200), StemPro MSC SFM (Gibco; cat. no. A1033201), StemFit For Mesenchymal Stem Cells (Ajinomoto), PRIME-XV MSC Expansion XSFM (Fuji Film; cat. no. 550-34201), and ADSC-4 (KOHJIN BIO; cat. no. 16030044). Cells were seeded in 2 mL medium at a density of 3 × 10^4^ cells/well on the dish without coating for Cellartis or StemFit culture or on vitronectin-N (Gibco; cat. no. A14700) coated dish for Stem Pro or CiMS culture. Cells were cultured for 4 days and passaged until the cells ceased to proliferate.

A coculture assay was conducted using MSCs (0.6 × 10^5^) from various culture conditions cultured together with PBMCs (lot no. 210972203C from a 23-year-old Caucasian female; lot no. 2107414001 from a 36-year-old Caucasian female; Lonza) at a 1:20 ratio. An MSC:PBMC coculture ratio of 1:20 was selected according to the following calculations. The total number of T cells per 60 kg body weight is approximately 1.2 × 10^10^ cells (for a 60 kg person, the blood volume is 4.8 L [80 mL/kg × 60 kg], the white blood cell count is approximately 3.0 × 10^10^ [average 6.0 × 10^3^ cells/mL × 4.8 × 10^6^ mL], and the T-cell percentage is 16%-34% [average 25%]), and the therapeutic dose of MSCs administered to a patient of 60 kg is reported to be 1.6-9.6 × 10.^8,20-22^ A PBMC vial contains approximately 60%-70% T cells.

The cells were incubated in RPMI 1640 medium (Thermo) for 24 h to evaluate acute reactions or for 72 h to evaluate subacute reactions. After stimulation of MSCs with PMA (Sigma-Aldrich; cat. no. P8139) and ionomycin (Sigma-Aldrich; cat. no. I0634), the concentrations of IL-2, IL-6, IL-10, IL-17, and TGFβ released in the coculture medium were measured by enzyme-linked immunosorbent assay (ELISA; Abcam) according to the manufacturer’s instructions. PBMCs were harvested to evaluate the induction of Tregs in the presence of MSCs under various culture conditions using flow cytometry (Aria II; BD, San Jose, CA, USA).

### Immunostaining and Flow Cytometry

Mitochondria in MSCs cultured either with CiMS or Cellartis were stained with AIE Mitochondria Red (AIEgen Biotech Co.) and observed by confocal microscopy (Olympus; FV1200) using a 40× objective lens. Mitochondrial membrane voltage and cell size were evaluated by flow cytometry (BD Aria II) after staining with AIE Mitochondria Red (AIEgen Biotech Co.). Surface antigens of MSCs were detected by flow cytometry after staining with anti-CD73 (eBioscience; cat. no. 17-0739-42), anti-CD90 (BioLegend; cat. no. 328121), and anti-CD105 (eBioscience; cat. no. 12-1057-42) antibodies in accordance with the supplier’s instructions. Tregs were detected by flow cytometry after harvesting PMBCs and staining with anti-CD3 (BD Biosciences; cat. no. 563798), anti-CD4 (BD Biosciences; cat. no. 561841), anti-CD8 (BD Biosciences; cat. no. 557750), anti-CD25 (BD Biosciences; cat. no. 560990), and anti-FOXP3 (BD Biosciences; cat. no. 560852) antibodies.

### Animal Study

All mouse experimental protocols were approved by the Animal Experiment Committee of the Foundation for Biomedical Research and Innovation at Kobe (20-03-03). SKG mice (BALB/cAJc1; CLEA Japan Inc., Tokyo, Japan) develop RA-like arthritis in the knee joints after administration of 20 mg mannan (Sigma-Aldrich; cat. no. M7504) intraperitoneally. At 6 weeks after administration of mannan, 1 × 10^6^ MSCs cultured under various conditions or phosphate-buffered saline (PBS) was administered via the tail vein. The immunosuppressive effects of the MSCs were examined by histological analysis of tissue sections from the limb joints of SKG mice at 2 weeks after administration of MSCs or PBS. Inflammatory reactions related to arthritis were evaluated according to the accumulation of mouse CD4^+^ cells, which were detected with anti-mouse CD4 antibodies (1:1000; Abcam; cat. no. ab183685), followed by Histofine Simple Stain MAX-PO(R) (Nichirei Biosciences Inc.; cat. no. 414341) as the secondary reagent, and immunoreactivity was visualized using the Liquid DAB+ Substrate Chromogen System (cat. no. K3468; Dako).

### Annexin V Staining

MSCs were harvested and resuspended at the density of 2 × 10^5^ cells/mL with the annexin-binding buffer. Then, 5 µL Annexin V Conjugate (Invitrogen; cat. no. A35110) was added to 100 μL of each cell suspension. Then, 400 µL annexin-binding buffer was added and the stained cells were analyzed using an ARIAII flow cytometer (BD) followed by analysis with FlowJo 10.8.1 (https://www.flowjo.com/).

### Cell Cycle Analysis

MSC suspensions (2 × 10^5^ cells/mL) were centrifuged at 300 × *g* for 5 min. Then, cells were resuspended with 500 mL PBS and 5 mL Cell Cycle Assay Solution Blue (cat. no. C549; DOJINDO, Japan). Cells were incubated at 37 °C for 15 min in the dark, passed through a cell strainer (cat. no. 352235; Falcon), and analyzed using a FACS ARIAII instrument (BD).

### X-gal Assay

MSCs (2 × 10^4^ cells/dish) were stained with Cellular Senescence Detection Kit-SPiDER-βGal (cat. no. SG03; DOJINDO), and 1 μL of 1 mg/mL Hoechst 33342 was added to stain nuclei. The cells were observed by confocal fluorescence microscopy (Olympus IX71; excitation: 488 nm, emission: 500-600 nm). The percentage of X-gal-positive cells was determined using Image J software.^30^ The average of the percentages of X-gal-positive cells from 3 different observation areas was scored for each culture condition.

### Ki67 Staining

MSCs were fixed on dishes with 4% paraformaldehyde overnight at 4 °C. The expression of Ki67 in cultured cells was detected with anti-Ki67 antibodies (1:100; cat. no. M72401; Dako). Binding was visualized with a secondary antibody labeled with Alexa Fluor 555 (1:500; cat. no. A21425; Invitrogen), and cells were counterstained with DAPI (cat. no. D1306; Life Technologies). The percentage of Ki67-positive cells was determined by visual observation.

### Mitochondrial Respiratory Assay

Mitochondrial functions were assessed by measuring the OCR (pmol/min) using the Seahorse XF Cell Mito Stress Test kit (Agilent). MSCs (1 × 10^4^) obtained from various culture conditions were cultured in 80 µL medium (CiMS or Cellartis) and placed on a Seahorse XF Cell Culture Microplate. Then, 2 µM oligomycin, 3 µM carbonyl cyanide 4-(trifluoromethoxy) phenylhydrazone, and 1 µM rotenone and antimycin A were added to the medium sequentially, and the OCR was measured using the Seahorse XFe/XF Analyzer (Agilent).

### Evaluation of Cell Morphology

The morphology of MSCs after 2 days of culture was captured using a microscope (Keyence; cat. no. BZ-X800). The pseudopods of MSCs were identified, and their total areas were calculated from 150 images acquired at 10× magnification using Cell Pocket ver. 2 (Shimadzu). The percentage of the pseudopod area relative to the total pseudopod area was divided by the total area of the MSCs.

### LC-MS/MS

The medium samples were analyzed using a triple quadrupole LC-MS/MS (LCMS-8050) and the Cell Culture Profiling Method Package (Shimadzu Corp) to determine the area ratio. The amounts of metabolites in the medium measured by LC-MS/MS were normalized to the number of cells harvested using the following formula: (area ratio − background)/cell number.

### Statistics

Two-way repeated measures ANOVA and Tukey post-hoc comparison tests were performed to compare IL-6 or IL-10 levels at 24 and 72 h between P3 and P8. Results with *P* values less than .05 were considered statistically significant. Results are expressed as means ± SDs.

## Supplementary Material

szad005_suppl_Supplementary_Material

## Data Availability

The data underlying this article will be shared on reasonable request to the corresponding author.
