# Supplementary material for: A Novel Approach for Determining the Critical Quality Attributes of Mesenchymal Stem Cells by Specifying Cell Population With Replication Potential
Source: Stem Cells Transl Med. 2023 Mar 14;12(3):169–82. doi: 10.1093/stcltm/szad005 (PMC10021496; doi:10.1093/stcltm/szad005)
Supplement: szad005_suppl_Supplementary_Material [file szad005_suppl_supplementary_material.pdf]

Fig. S1

MSC-1(adipocyte origin)

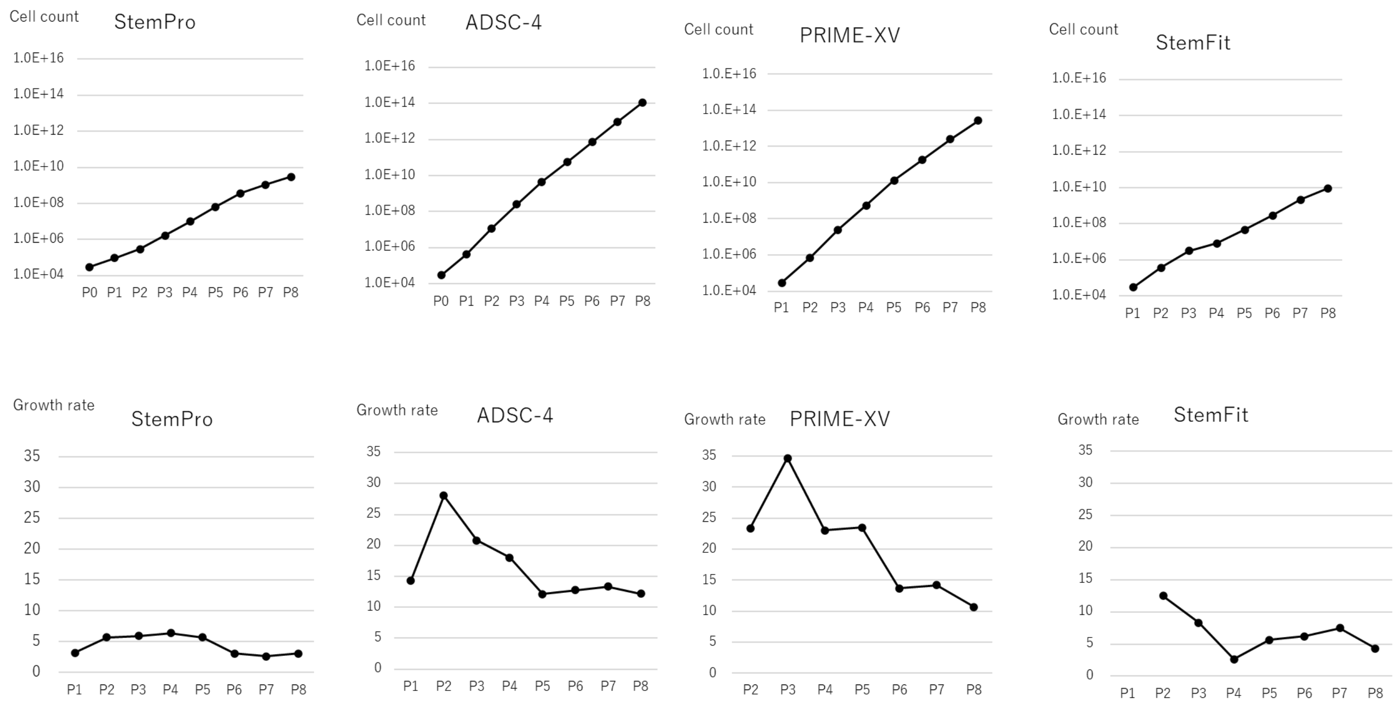

MSC-4 (adipocyte origin)

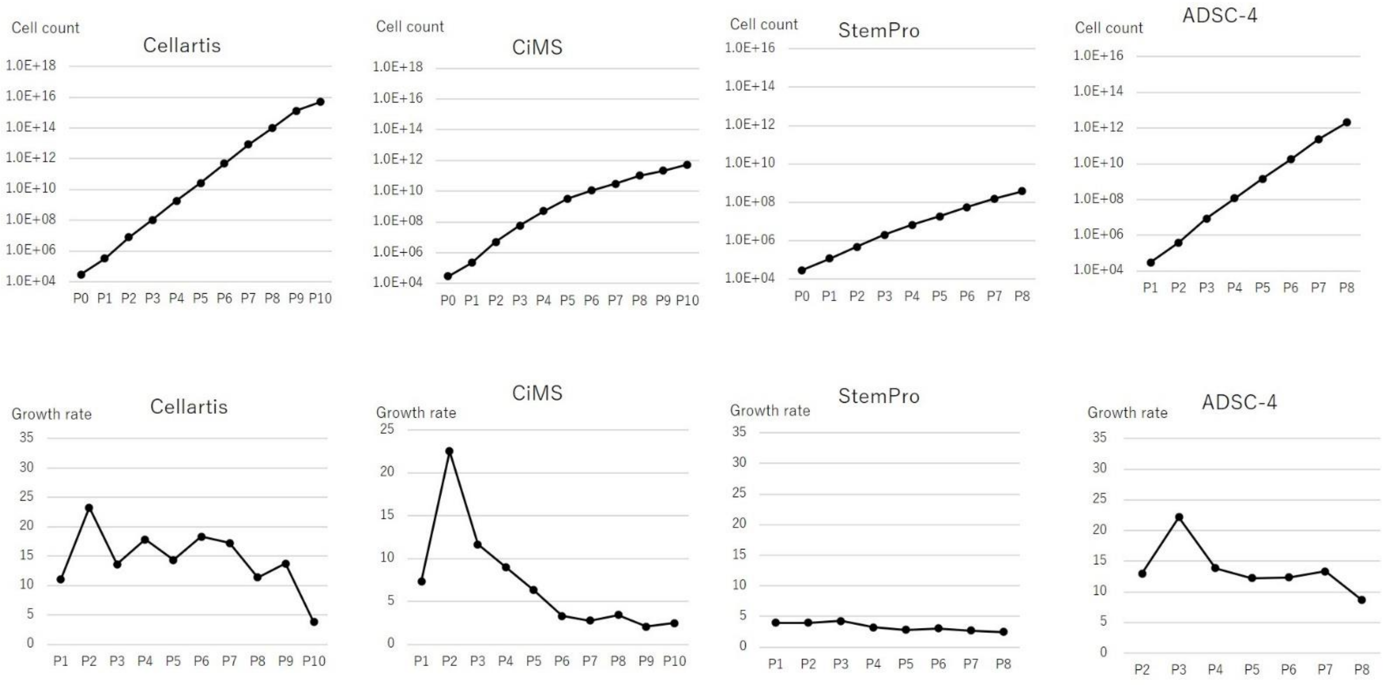

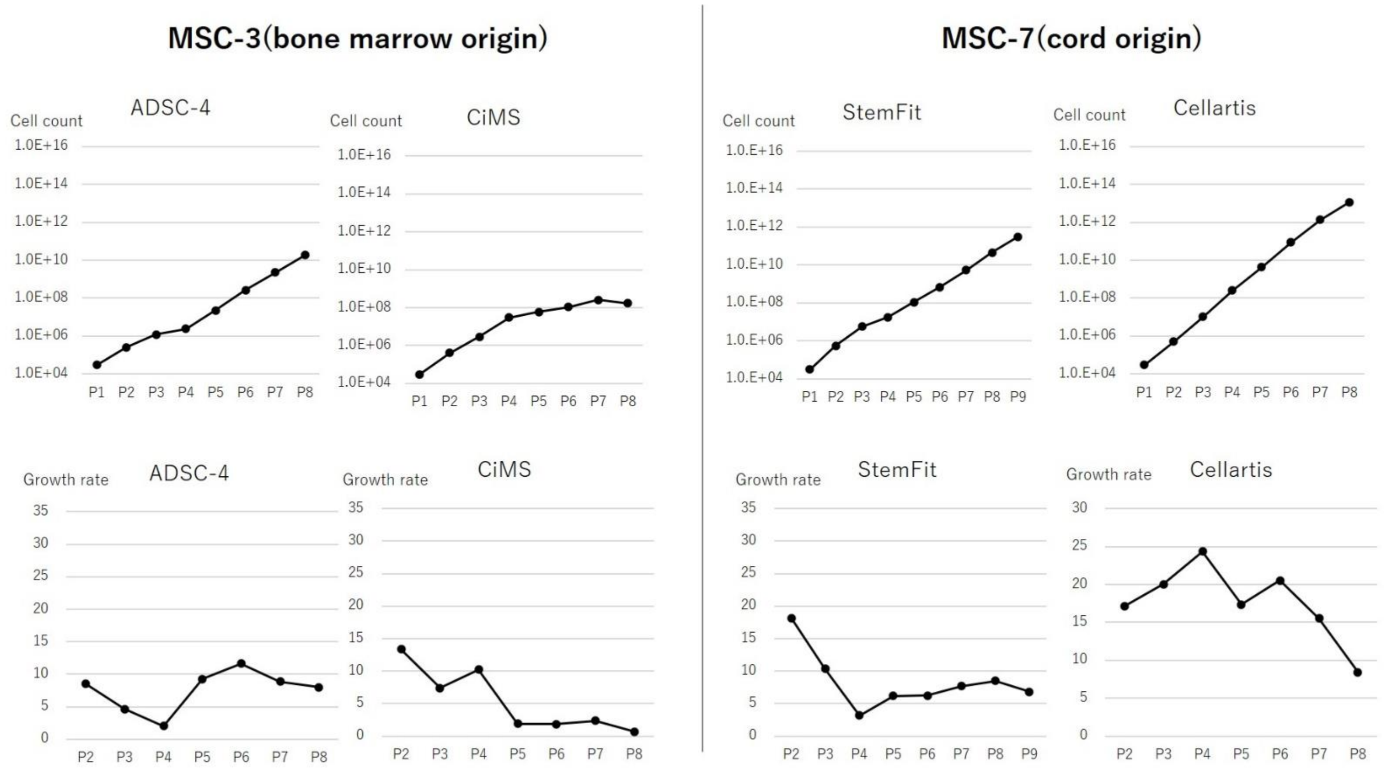
MSC-3(bone marrow origin)
MSC-7(cord origin)
Cell count
ADSC-4
CiMS
StemFit
Cellartis
1.0E+16
1.0E+14
1.0E+12
1.0E+10
1.0E+08
1.0E+06
1.0E+04
1.0.E+16
1.0.E+14
1.0.E+12
1.0.E+10
1.0.E+08
1.0.E+06
1.0.E+04
P1 P2 P3 P4 P5 P6 P7 P8
P1 P2 P3 P4 P5 P6 P7 P8 P9
Growth rate
35
30
25
20
15
10
5
0
P2 P3 P4 P5 P6 P7 P8
P2 P3 P4 P5 P6 P7 P8 P9

Fig. S2

MSC-1 (adipocyte origin)

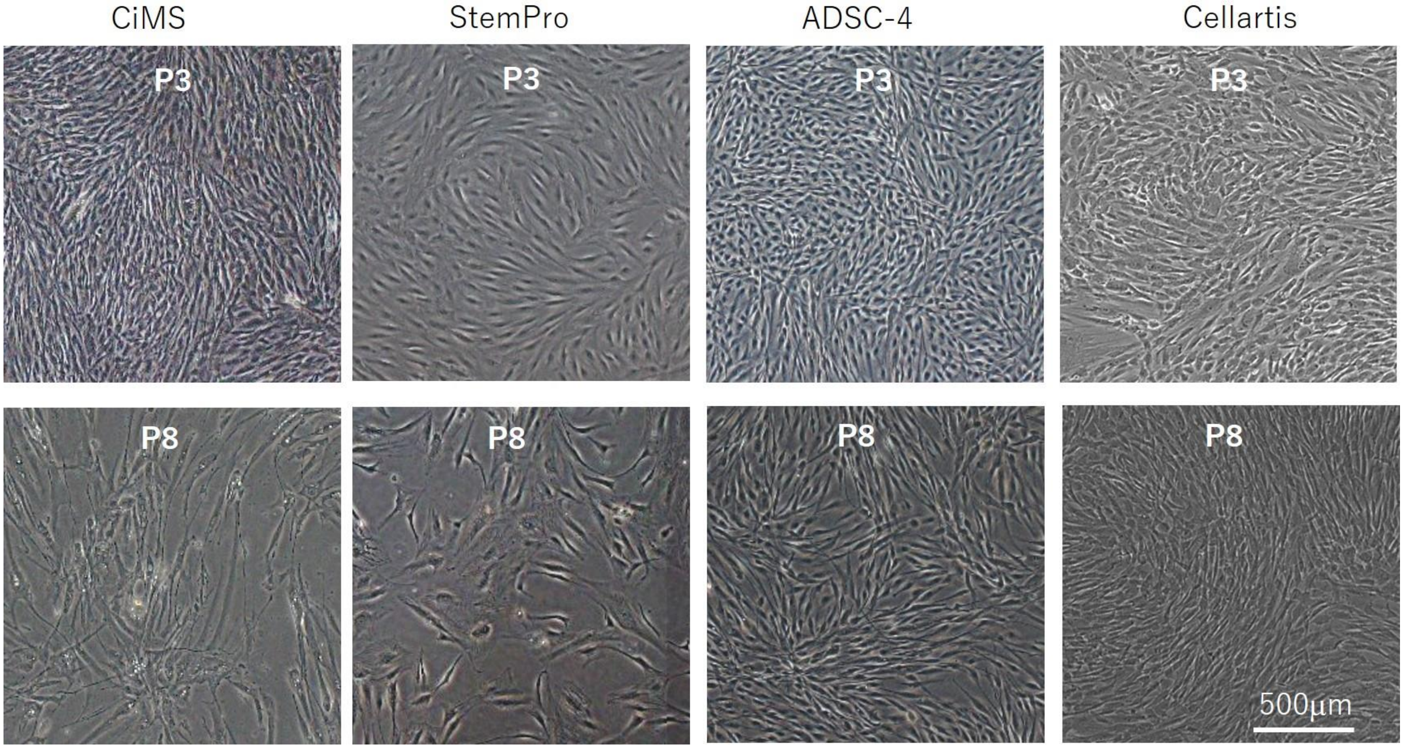

MSC-4 (adipocyte origin)

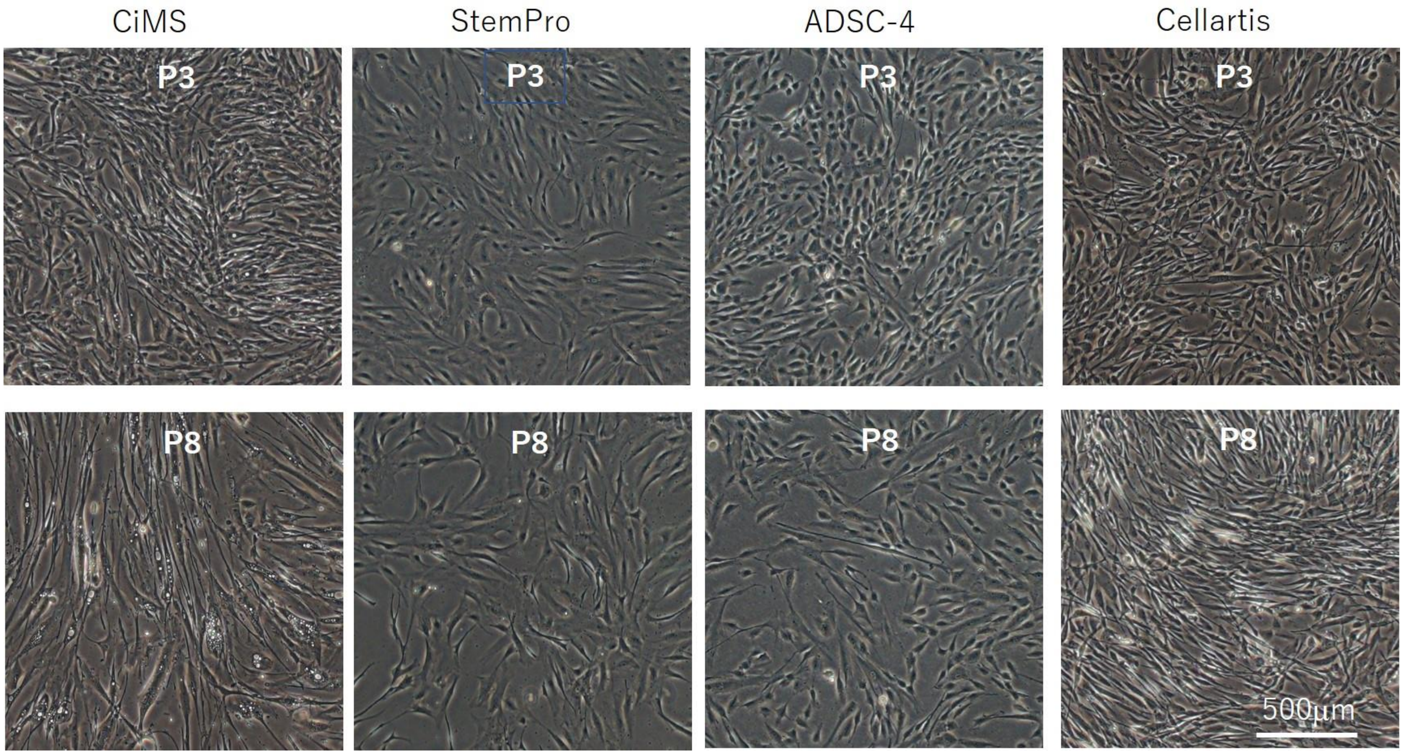

Fig. S3

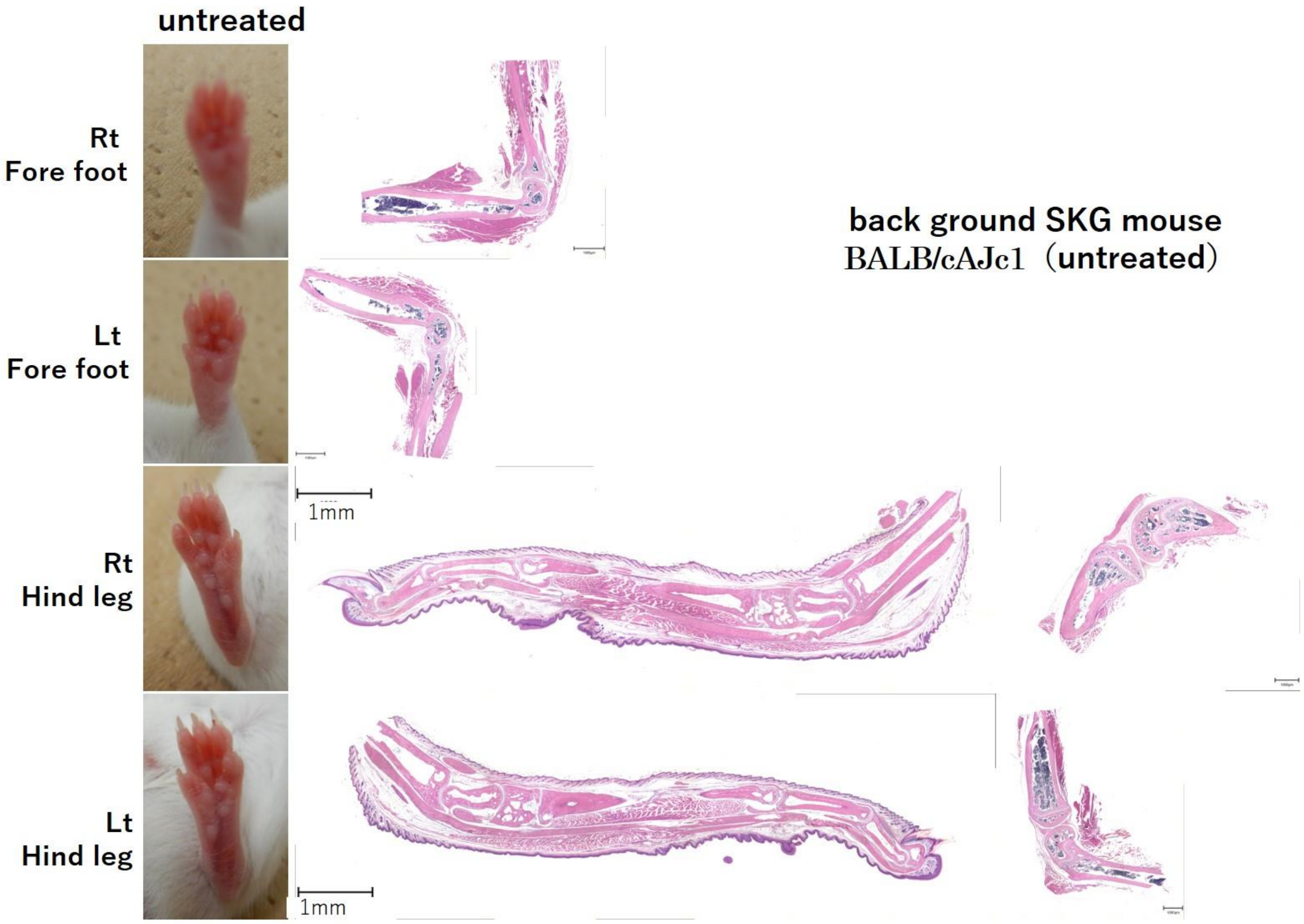
untreated
Rt
Fore foot
Lt
Fore foot
Rt
Hind leg
Lt
Hind leg
1mm
1mm
back ground SKG mouse
BALB/cAJc1（untreated）

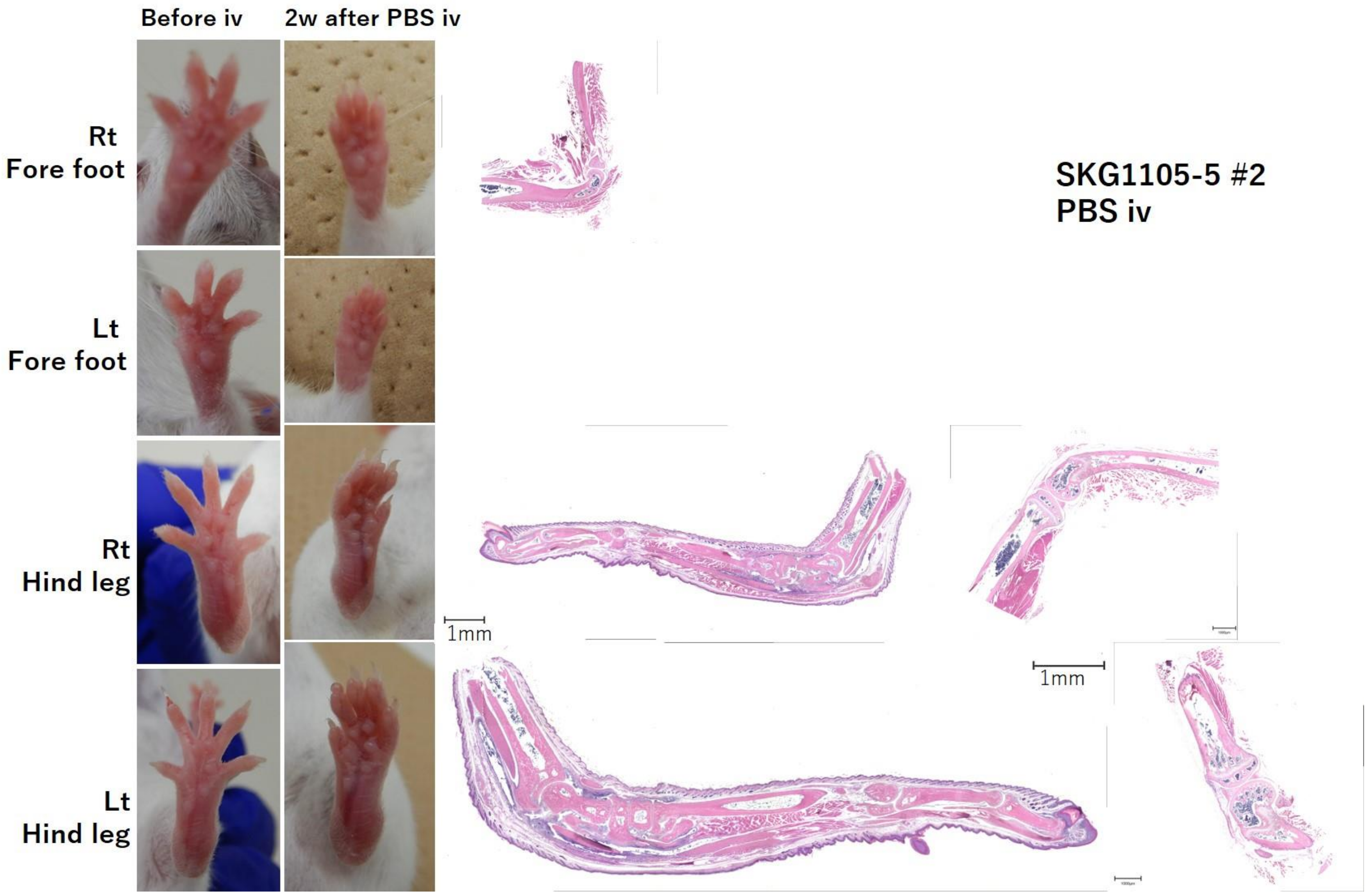
Before iv
2w after PBS iv
Rt
Fore foot
Lt
Fore foot
Rt
Hind leg
Lt
Hind leg
1mm
1mm
SKG1105-5 #2
PBS iv

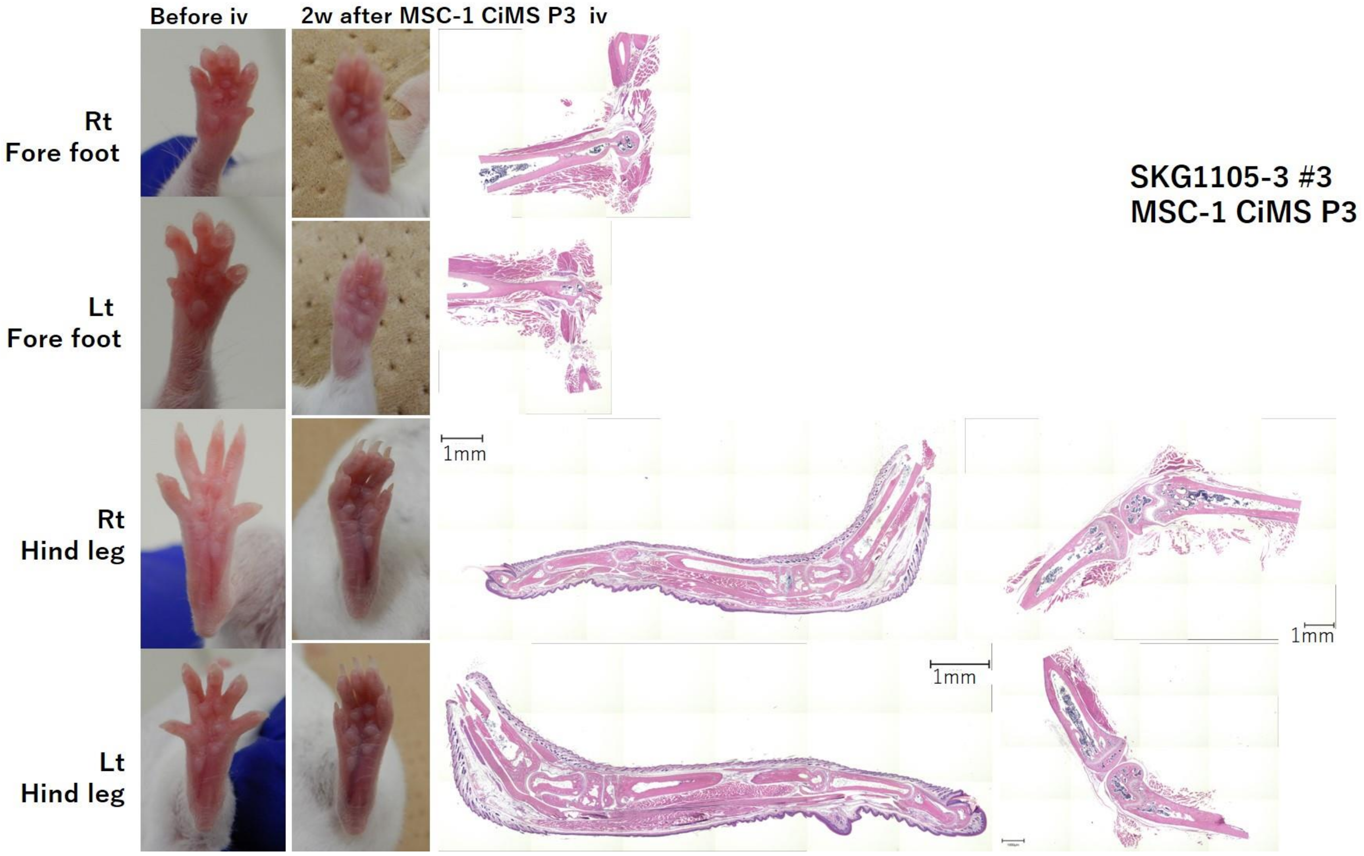
Before iv
2w after MSC-1 CiMS P3 iv
Rt
Fore foot
Lt
Fore foot
Rt
Hind leg
Lt
Hind leg
1mm
1mm
1mm
SKG1105-3 #3
MSC-1 CiMS P3

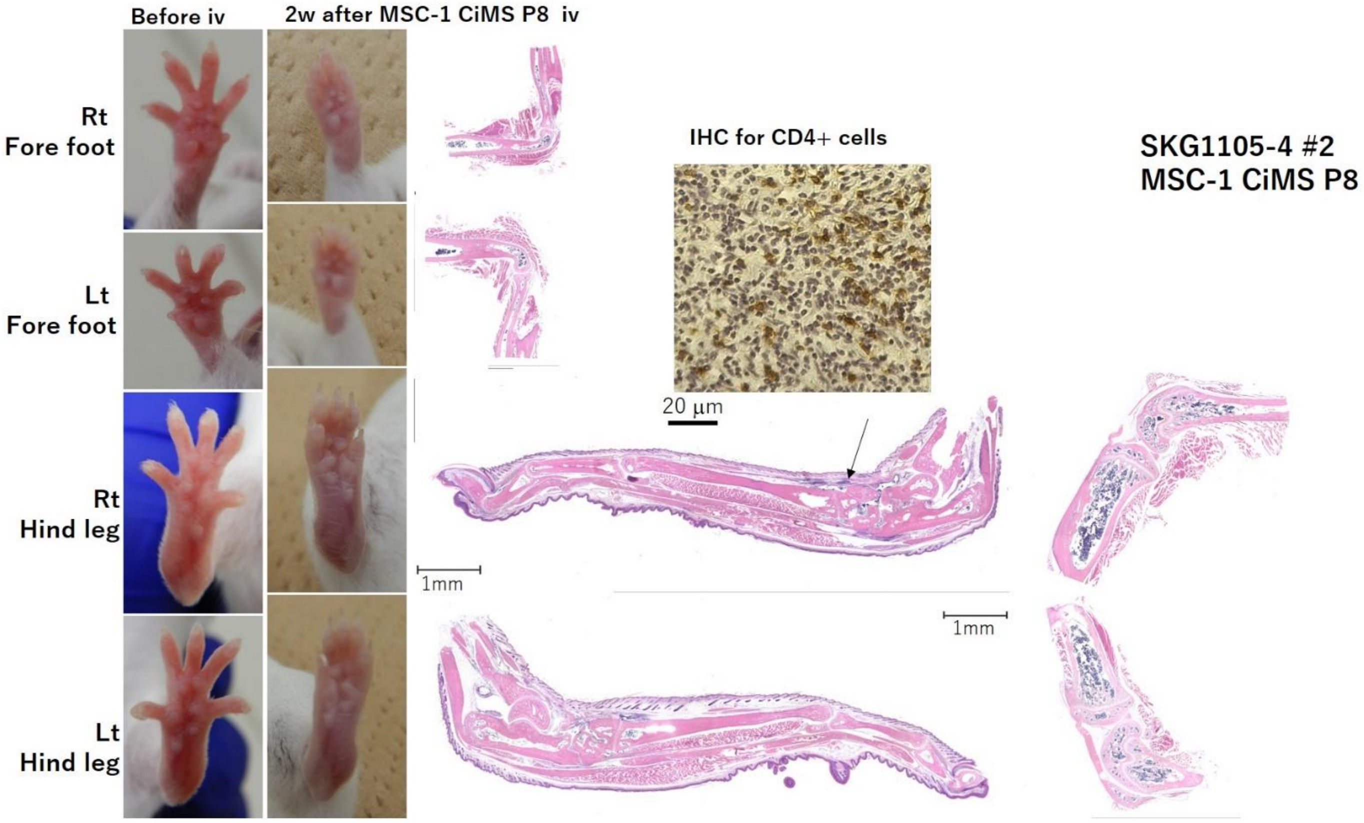
Before iv
2w after MSC-1 CiMS P8 iv
Rt
Fore foot
Lt
Fore foot
Rt
Hind leg
Lt
Hind leg
IHC for CD4+ cells
20 µm
1mm
1mm
SKG1105-4 #2
MSC-1 CiMS P8

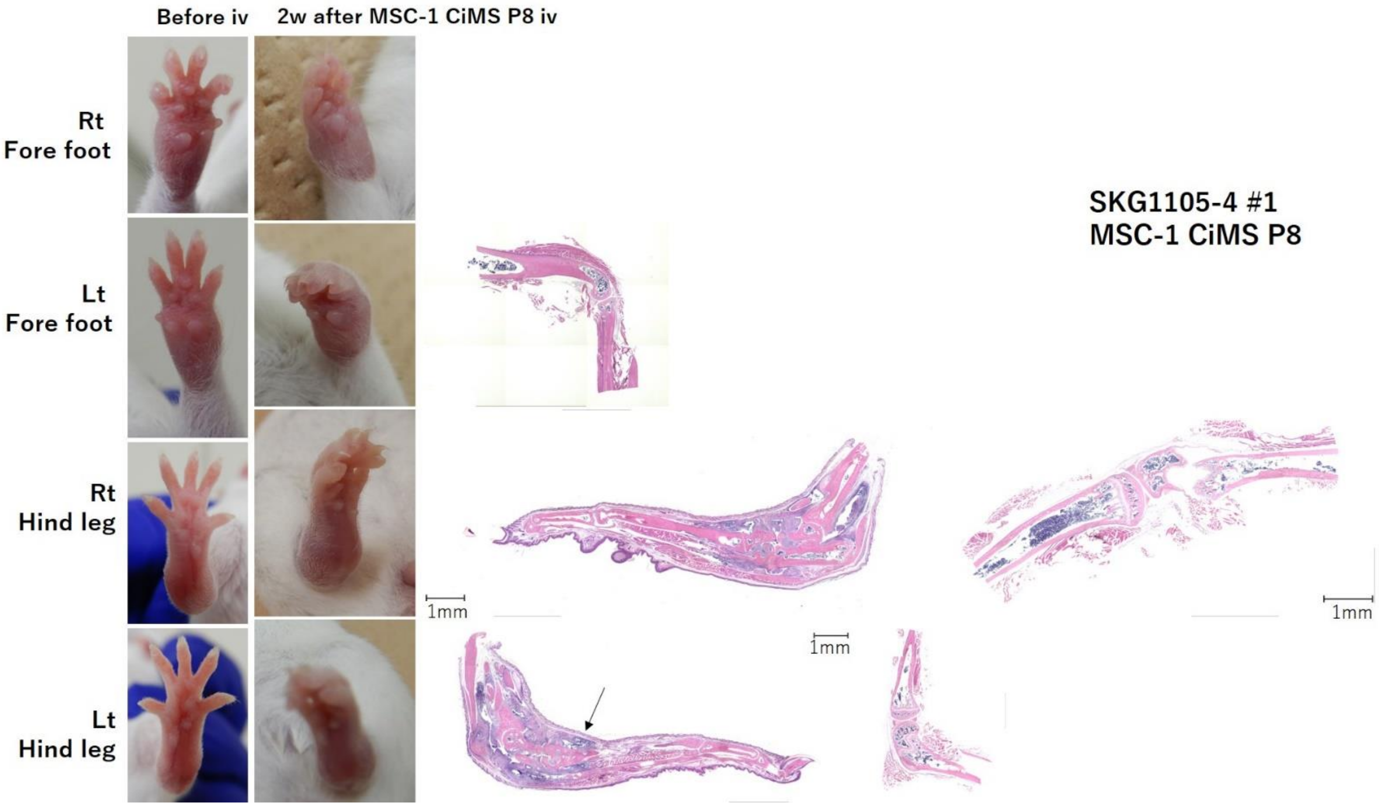
Before iv
2w after MSC-1 CiMS P8 iv
Rt
Fore foot
Lt
Fore foot
Rt
Hind leg
Lt
Hind leg
SKG1105-4 #1
MSC-1 CiMS P8
1mm
1mm
1mm

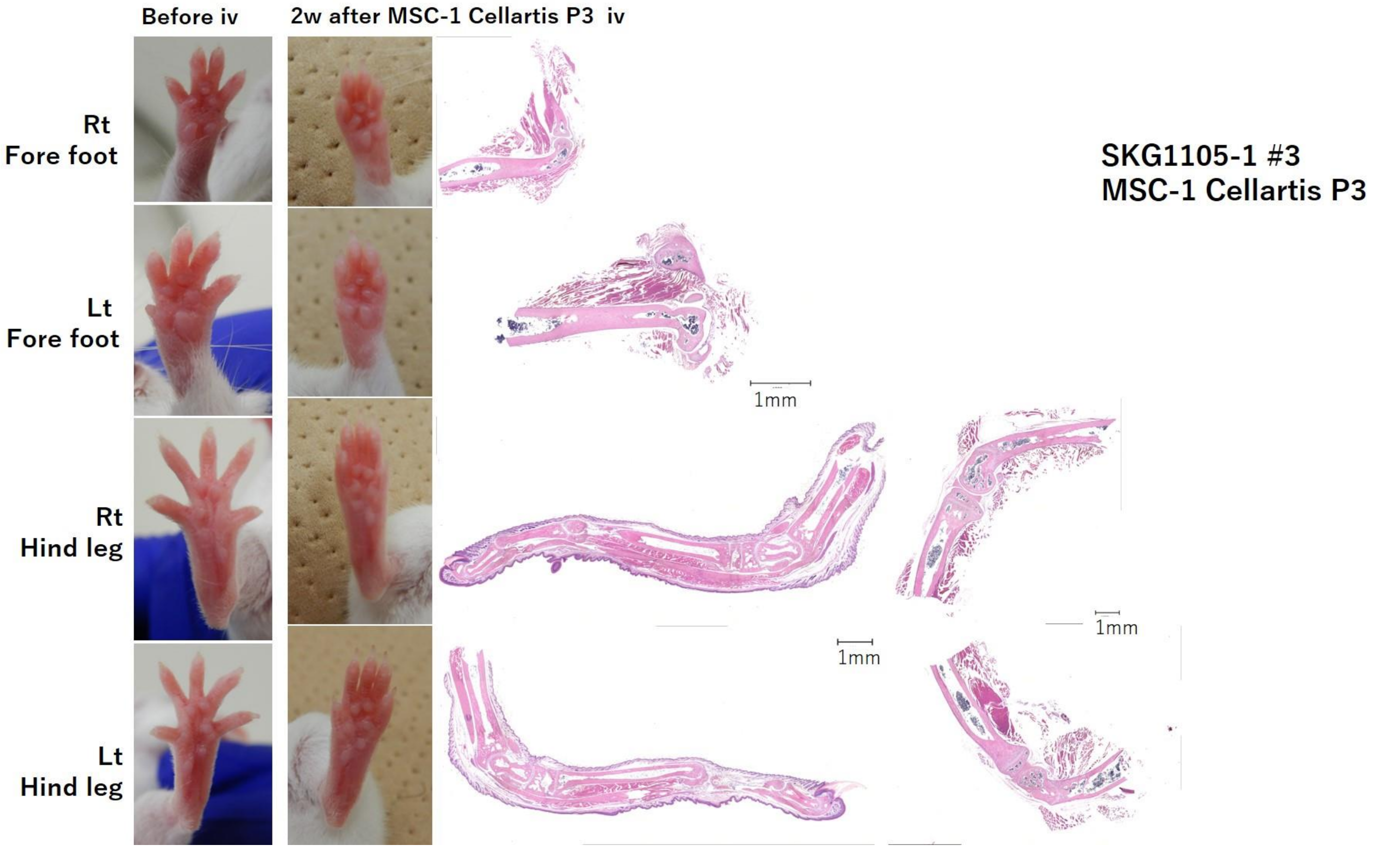
Before iv
2w after MSC-1 Cellartis P3 iv
Rt
Fore foot
Lt
Fore foot
Rt
Hind leg
Lt
Hind leg
SKG1105-1 #3
MSC-1 Cellartis P3
1mm
1mm
1mm

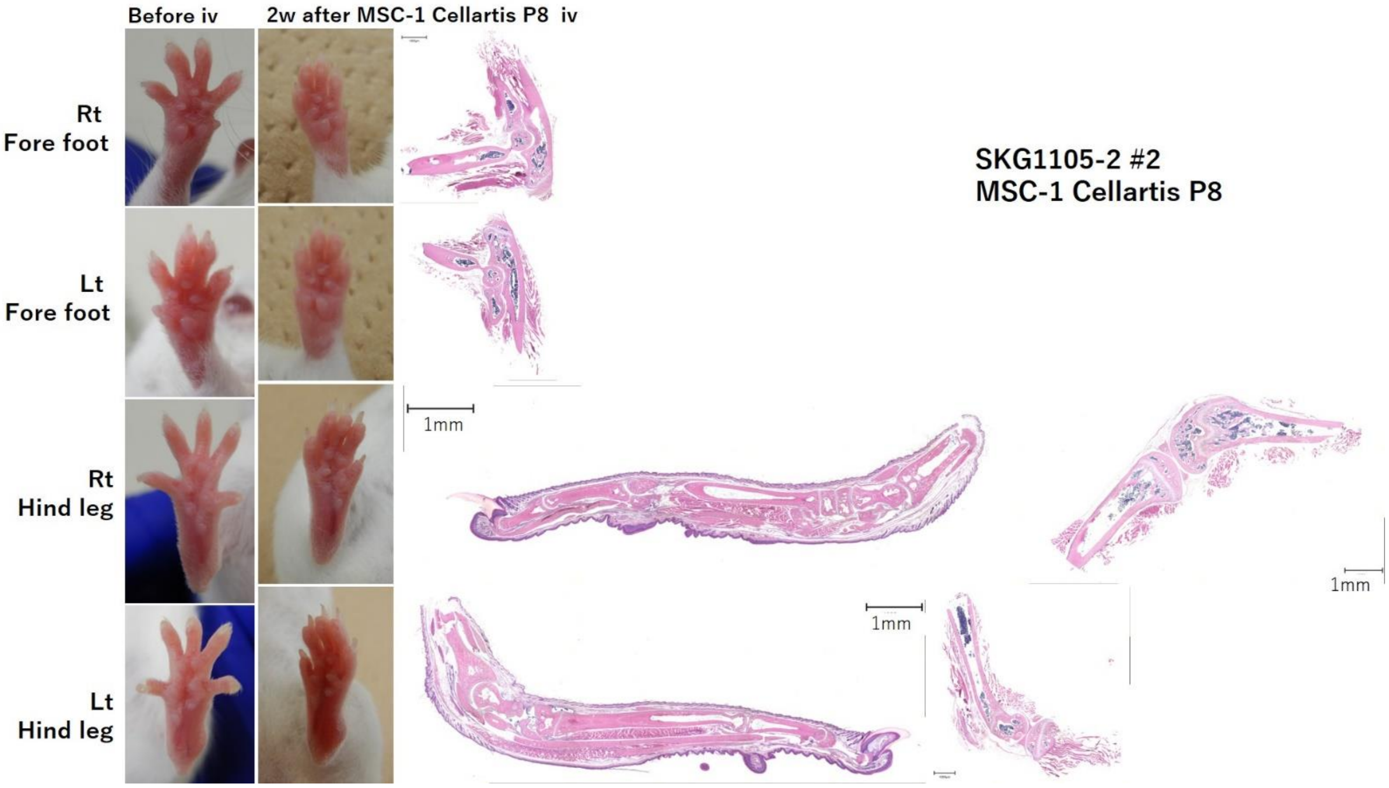

Before iv
2w after MSC-1 Cellartis P8 iv
Rt
Fore foot
Lt
Fore foot
Rt
Hind leg
Lt
Hind leg
1mm
1mm
1mm
SKG1105-2 #2
MSC-1 Cellartis P8
